# Area-based units of analysis for strengthening health inequality monitoring

**DOI:** 10.2471/BLT.15.165266

**Published:** 2016-08-31

**Authors:** Ahmad Reza Hosseinpoor, Nicole Bergen

**Affiliations:** a World Health Organization, avenue Appia 20, 1211 Geneva 27, Switzerland.; b Faculty of Health Sciences, University of Ottawa, Ottawa, Canada.

Inequalities in health persist worldwide and one of the starting points for remedial action is collecting data that reveal patterns of inequality. Current discussions about the best ways of monitoring health inequalities emphasize disaggregating data by variables such as socioeconomic status, geographical area or sex. The sustainable development goals (SDGs) adopted in 2015 include a call for countries to increase the availability of disaggregated data as part of the aim to strengthen data monitoring and accountability (SDG target 17.18).^1^ Yet countries have varying capacities for monitoring health inequality. This is due in part to data-related issues such as weaknesses in the health information systems, especially in many low- and middle-income countries; lack of availability or poor quality of health data; and a limited ability to disaggregate data across all health topics within countries.^2^ Overcoming these challenges in the long term requires substantial investments in the health information infrastructure.^3^^,^^4^ In the short-term, countries need innovative approaches to best harness the potential of their existing data to improve monitoring efforts.

Current approaches to health inequality monitoring tend to focus on data collected through household health surveys. These provide two streams of data – about health indicators and about the dimensions of inequality – at the individual or household level. This makes such surveys the main source of data for within-country monitoring of health inequality especially in low- and middle-income countries. However, household health surveys have certain limitations. In many low- and middle-income countries they tend to cover only a narrow set of topics, such as reproductive, maternal, newborn and child health. Other health topics, such as infectious diseases or road traffic injuries, are rarely the focus of household surveys. Household health surveys and their consequent reporting tend to be done outside the regular activities of the health information system, and are resource intensive. Furthermore, data from household surveys may not be representative of small subpopulations of interest, and so cannot be used for certain purposes, such as assessing cross-district inequality, due to too small sample size at that administrative level. By increasing the use of area-based units of analysis, including greater integration of data from other reliable data sources – including vital registration systems, censuses and administrative data – the possibilities for health inequality monitoring may be strengthened and expanded across health topics.

In this article we make the case for stratifying data at the level of subnational geographical regions, such as provinces, states or districts. The wider use of an area-based unit of analysis as a complementary way to analyse data at the individual or household level has certain practical advantages that are relevant to low- and middle-income countries as well as high-income countries.

First, this approach opens up new possibilities concerning the data that can be used for within-country monitoring, in terms of both health data and data about dimensions of inequality. In some cases, individual or household data on both health and inequality dimensions may be unavailable in one data source; if these data were available from different data sources (e.g. those that collect data at the level of subnational regions), alternative ways of capturing area-level estimates may provide an insight into the extent of inequality. For instance, whereas data about economic status, race, ethnicity, migratory status or disability may not always be collected alongside health data at an individual or household level, they may be available by region. Subnational regions are often aligned with administrative districts, which facilitates the use of administrative-level data. For example, the distribution of health system inputs and outputs (e.g. service delivery) can be compared to health determinants (e.g. district-level poverty, education or employment).

Second, since interventions to reduce inequities are likely to be implemented at the local administrative level, regional monitoring of health inequalities may be a useful tool for benchmarking, with implications for resource allocation, planning and evaluation. This is particularly true when a country’s health system administration is decentralized because substantial differences may exist across geographical areas.^5^

Third, area-based measures may provide a more intuitive understanding of health inequalities and may help to identify possible points for intervention. Geographically defined subpopulations are by nature easy to identify and locate, and health interventions may thus be effectively targeted to disadvantaged regions.^6^ For example, measuring health inequality on the basis of household wealth using asset-based indices may pose limitations in terms of identifying and reaching disadvantaged subpopulations, as the poorest segment of the population may be located throughout different regions of a country.^7^^,^^8^

Alongside these advantages, some caution is needed when adopting an area-based unit of analysis. There is the risk of committing a so-called ecological fallacy (i.e. making assumptions about individuals based on population-level patterns, or in this case, erroneously drawing conclusions about the health of individuals using area-based data). For instance, if richer districts were found to have a higher prevalence of road traffic injuries it could not be assumed that road traffic injuries are more prevalent among richer individuals. Also, ethical concerns surrounding the confidentiality of data for individuals and communities may arise if secondary data from existing sources such as censuses or health records are linked to other data sources. (Linking data refers to the use of small-area identifiers to associate data between different sources, such as using postcodes as a way to link health records and area-level socioeconomic deprivation.) These ethical issues may be addressed through measures to respect personalized data through anonymization, and abiding by guidelines of ethical review boards and data privacy bodies.

Actions to increase the quality and availability of area-based data include standardizing the data collected by health facilities, and adopting electronic data collection and storage methods. Common systems of small area coding can be applied across data sources – such as censuses, civil registration and vital statistics, surveys, and facility data – permitting linkages between different sources. This practice of linking between data sources is currently more common in high-income countries than low- or middle-income countries.

Two distinct applications of an area-based analysis of within-country inequality are: (i) measuring area-based socioeconomic inequality and (ii) measuring regional inequality per se. The differences between these two approaches have implications for the measurement and interpretation of health inequality (Table 1).

**Table 1:** Contrasting features of two approaches to adopting an area-based unit of analysis for monitoring health inequality

Approach	Basis of inequality	Characteristics of subgroups	Data source characteristics	Applicable measures of inequality
Area-based socioeconomic inequality	Socioeconomic characteristics ascribed to region	May be ranked by socioeconomic status	Data may be obtained from a single source or multiple sources (if there is an identifier that serves as a link between the sources)	Pairwise measures
				Concentration index
				Slope index of inequality
Regional inequality per se	Geographical region	Inherently unordered	Data are typically obtained from a single source	Pairwise measures
				Index of disparity
				Theil index
				Mean difference from mean

In the first case, a socioeconomic characteristic (e.g. median income, median level of education, deprivation index, employment rate) is ascribed to a region. Geographical units, such as municipalities, are assigned a socioeconomic characteristic, such as median income of the municipal population. In this example, the municipalities can be logically expressed as an ordered variable (e.g. ranked by wealth), permitting calculations such as simple pairwise measures (i.e. difference and ratio of richest to poorest municipalities), concentration index and slope index of inequality.^3^ In addition, disaggregated data can be inspected to identify characteristic patterns of inequality.^3^

To facilitate analyses of socioeconomic inequality using a geographic unit, several deprivation indices have been constructed and applied to small geographical areas, such as census tracts, electoral wards, postcode areas or municipalities. These indices encompass several factors, such as income, employment, housing, crime, education, access to services and living environment.^9^^,^^10^ When health data contain a corresponding identifier, linkages may permit the use of multiple data sources. Previous research has measured area-based socioeconomic inequality in the prevalence of about 40 different types of morbidity.^11^ Clinical data from a database of individuals’ medical records were linked at the postcode level to socioeconomic status, which was determined using the Carstairs deprivation index (based on unemployment, overcrowding, car ownership and low social class); postcodes were grouped into deciles according to their ranking.

In the second case, the measurement of subnational regional inequality per se is based on comparisons between geographically defined regions, independent of any descriptive characteristics of these regions. Since geographically defined regions are inherently unordered and have no natural ranking, certain measures used to express socioeconomic inequality may not be appropriate. Alternative measures should be considered, such as the mean difference from overall mean, the index of disparity or the Theil index,^12^ along with measures that apply in both scenarios, such as simple pairwise measures.^3^ A study of health inequality within four countries on the basis of regional inequality per se demonstrated inequality in reproductive, maternal, newborn and child health indicators by subnational region.^12^ In addition to presenting disaggregated data, the study compared the suitability of several measures of inequality.

In many countries, health inequality monitoring systems could be strengthened by expanding the capacity for, and practice of, area-based health inequality monitoring. Adopting an area-based unit to express health inequality has several merits. Area-based units of measurement provide a clear way to identify disadvantaged subgroups, and provide an effective evidence base for designing targeted interventions. Furthermore, administrative data can be harnessed to provide inputs for health inequality analyses, and may offer additional insight into how regional factors are associated with health. Monitoring health inequalities by geographically defined subgroups can help to identify disadvantaged regions that are falling behind in terms of health indicators and to guide improvements in these areas.
